# Bayesian VARs of the U.S. economy before and during the pandemic

**DOI:** 10.1007/s40822-023-00229-9

**Published:** 2023-04-08

**Authors:** Anna Sznajderska, Alfred A. Haug

**Affiliations:** 1 Warsaw School of Economics, Al. Niepodległości 162, 02-554 Warsaw, Poland; 2 Department of Economics, University of Otago, Dunedin, 9054 New Zealand

**Keywords:** COVID-19, Bayesian VAR models, Impulse response functions, Forecasting, C32, E32, F10, O50

## Abstract

We compare the forecasting performance of small and large Bayesian vector-autoregressive (BVAR) models for the United States. We do the forecast evaluation of the competing models for the sample that ends before the pandemic and for the sample that contains the pandemic period. The findings document that these models can be used for structural analysis and generate credible impulse response functions. Furthermore, the results indicate that there are only small gains from the application of a large BVAR model compared to a small BVAR model.

## Introduction

Vector Autoregressions (VARs) are one of the most popular tools within central banks and academia to analyze and forecast economic developments. The VAR models provide a very general representation of a statistical model, often in linear form. VARs allow the capture of complex dynamic data relationships, because in contrast to other models, such as structural economic models, they do not impose economic restrictions on the parameters.^1^ Broadly speaking, the variables in the VAR model depend on their own lags and other model variables’ lags.

The problem is that the high level of generality implies a large number of parameters to be estimated, whereas the typical sample size for macroeconomic applications is rather small. This entails a risk of overparameterization, resulting in parameter instability and a loss of precision for forecasting. On the other hand, reducing the number of parameters by cutting back on variables in a VAR may potentially lead to an omitted variable bias with adverse consequences both for structural analysis and forecasting. Furthermore, frequently too many lags are included in a VAR model to improve the in-sample fit, which results in a significant loss of degrees of freedom and poor out-of-sample forecast performance.^2^

The recent literature has proposed a number of ways to overcome these problems. There are three main econometric recommendations. The first is to apply factor models, which are based on the assumption that interrelations within a large dataset can be explained by a few common factors. The second is to use high frequency data, which may be used to identify the shocks from outside the model. The third is to take the Bayesian point of view, because the supply of prior information limits the overparameterization issue.^3^

The aim of this paper is to assess the performance of the Bayesian VAR (BVAR) for monetary models during the COVID-19 pandemic. The idea is to model the pandemic shocks and capture the adjustment process of the economy through the BVAR model parameters, without imposing a structural change in those parameters. Assuming in contrast a structural break would imply that the model parameters have changed due to the pandemic and that economic relationships are different after the start of the pandemic. A serious drawback of such conventional breaks is that they are treated as having zero probability of occurring again in the future and are thus ignored in forecasting (Hamilton, 2016). Instead, we use relatively flexible and not very restrictive formulations of the statistical distributions for the regression parameters and errors to capture the pandemic shocks, as well as the shocks due to the recent global financial crisis. The statistical tool we use for that purpose is Bayesian modeling.

This paper formulates and estimates small and large BVAR models for the U.S. economy before and during the pandemic. It contributes to the recent literature on BVARs that proposes ways to deal with the COVID-19 episode in macro-econometric VAR models. Schorfheide and Song (2021) drop observations during the early part of the pandemic (March – June 2020) in a mixed frequency BVAR with monthly and quarterly observations. Lenza and Primiceri (2022), Carriero et al. (2022), and Bobeica and Hartwig (2022) account in alternative ways for the excess variability of different macroeconomic series during the pandemic period. Lenza and Primiceri (2022) inversely weigh the observations during the pandemic period based on the innovation variances, using a standard homoskedastic VAR. On the other hand, Bobeica and Hartwig (2022) use a multivariate t-distribution for errors in a small-scale BVAR. Carriero et al. (2022) use a mid-sized BAVR with variable-specific outlier-adjusted stochastic volatility and t-distributed innovations. The idea behind using a distribution with fatter tails, i.e., the t-distribution, is to capture large shocks, such as those occurring during the recent pandemic. Instead, we use a recently proposed flexible Bayesian methodology for small- and large-scale VARs, developed and successfully applied in the pre-pandemic period to forecasting U.S. macroeconomic time series by Giannone et al. (2015).^4^ In other words, we explore whether a BVAR model used for forecasting with good results before the pandemic is able to forecast well once the pandemic period is included, without dropping observations, outlier adjustments, or assuming a t-distribution.

Giannone et al. (2015) build on the approach in Bańbura et al. (2010) that formulates a BVAR based on the conjugate normal-inverse Wishart family for the variance–covariance matrix and the VAR coefficients. To estimate the model, our paper uses the BEAR toolbox of the European Central Bank (Dieppe & van Roye, 2021; Dieppe et al., 2016). Our paper divides the variables into fast-moving financial variables and slow-moving real variables and prices, with the identifying assumption that the slow-moving variables do not respond contemporaneously to a monetary policy shock and the information set of the monetary policy contains only past values of the fast-moving variables. We consider a small and a large BVAR model. The small model includes industrial production, the consumer price index and a measure of the Federals Funds Rate. The large model includes employment, housing and other financial indicator data. Next, we conduct impulse response function and forecasting analyses with samples that include and exclude the pandemic.

Section 2 presents a short review of related Bayesian research. Section 3 introduces the basic features of our BVAR model and the data employed. Section 4 discusses the results and Sect. 5 concludes.

## A brief review of recent related literature

An important application of VAR and BVAR models in economics has been in the area of forecasting the future path of variables. Also, VAR and BVAR models have been used extensively for studying how structural economic shocks, such as fiscal and monetary policy shocks, affect the macro-economy (output, employment, inflation, interest rates, etc.). VAR and BVAR methodologies are an essential ingredient for economic model building and for guiding economic policy.

Petropoulos et al. (2022) survey forecasting theory and practice. They cover various alternative methods of forecasting from autoregressive moving-average models to state space, dynamic stochastic general equilibrium, Markov-switching and Bayesian models, as well as neural network and machine learning models.^5^ The various models impose different statistical and economic restrictions (i.e., assumptions) to make them operational for forecasting. Besides, their survey includes a vast range of forecasting applications in practice for a range of disciplines, such as climate science, demographics, energy, health, sports, supply chain management, and macroeconomics. More details on VARs and BVARs in macroeconomics and their origins can be found in Christiano (2012), focusing on contributions of Christopher A. Sims and the literature building on Sims’ work. The idea for VARs and BVARs in forecasting is to let the data talk as much as possible by imposing a minimal set of economic and statistical restrictions, in comparison to other available alternative statistical methods and economic models. The VAR allows for rich dynamics among the variables without economic restrictions on the VAR coefficients. Also, an important advantage of VARs over some other forecasting models is that variables in a VAR are treated as endogenous so that contemporaneous feedback among variables is allowed for. The statistical model is typically in linear or log-linear form with Gaussian error-terms. A further useful source in this area is Kilian and Lütkepohl (2017), discussing merits and limitations of VAR-based methodologies.

Impulse response function analysis studies how an economic shock affects the variables in the model over time, ceteris paribus. The VAR and BVAR models are reduced-form representations and their estimation-residuals have no economic interpretation. In order to carry out impulse response function analysis, structural shocks with economic meaning and interpretation are needed. To link the reduced form models to their structural versions one needs to make some economic assumptions in order to be able to identify structural shocks, such as monetary policy shocks. The literature has suggested various ways to do this, from restricting some structural shocks to not affect other variables contemporaneously (exclusion or zero short-run restrictions) and hence having effects only after a one-period (one month in our case) delay; long-run restrictions; sign restrictions; variance–covariance restrictions; restrictions extraneous to the model; or a combination of those (Christiano et al., 1999; Kilian & Lütkepohl, 2017). For example, sign restrictions require specifying periods affected by the restrictions, along with other assumptions that may make it difficult to recover a unique model with the true impulses because impulse responses are only set-identified (see, e.g., Fry & Pagan, 2011). Instead, we will follow in this article a relatively simple Cholesky-based block-triangular identification scheme in order to see whether it can produce reasonable impulse responses before and during the pandemic period.^6^

While BVAR models started out with small models, recent research has developed ways to deal with large BVAR models (Bańbura et al., 2010, Kapetanios et al., 2012, Bobeica & Jarociński, 2019, and Crump et al., 2021). Our econometric approach is based primarily on the work of Bańbura et al. (2010). They describe a large BVAR model with Bayesian shrinkage as an appropriate tool for forecasting and structural analysis. Their dataset covers the period 1959:01 to 2003:12 and hence does not include developments in regards to the pandemic. Also, new shrinkage methods, such as the procedure of Giannone, Lenza and Primiceri that is developed in Giannone et al. (2015), were introduced after the publication of their article.

A contemporary paper on large BVAR models is Crump et al. (2021). The paper is centered around the concept of conditional forecasting. The authors use quarterly data from 1973:Q1 to 2018:Q3. It is worth noting that our paper considers unconditional forecasting instead and uses monthly data between 1990:01 and 2021:11. Crump et al. (2021) define the impulse response functions as the difference between conditional and unconditional forecasts. They compare their forecasts with those of professional forecasters and Greenbook forecasts. The authors conclude that a large-scale BVAR model produces reliable predictions of the joint distribution for a large set of macroeconomic and financial indicators monitored by the Federal Reserve staff and professional forecasters.

We briefly describe a few papers closely related to our research. Carriero et al. (2011) compare the forecasting performance of numerous BVAR models. Their general finding is that there are only very small losses from the adoption of BVAR modeling choices that make forecast computation quick and easy. An approach that works well is to specify a normal-inverted Wishart prior along the lines of Sims and Zha (1998) for the VAR in levels, preferably optimizing its tightness and lag length. Also they argue that specifications in levels benefit substantially from the imposition of the sum-of-coefficients and dummy initial observation priors.

Kapetanios et al. (2012) apply a large BVAR model (with 43 variables), as one of three different alternative models, to examine the effects of unconventional monetary policy in the UK.^7^ The BVAR model is estimated over rolling windows to allow for structural change. Their work is based on the assumption that quantitative easing (QE) reduced medium- to long-term government bond yields by about 100 basis points in the UK. They estimate the effects of QE on real GDP and inflation using counterfactual scenarios. The results show that without QE real GDP and inflation would have fallen even more during 2009.

Several recent papers deal with the COVID-19 pandemic in BVAR models, such as Lenza and Primiceri (2022) and Bobeica and Hartwig (2022). Lenza and Primiceri (2022) apply the BVAR model with observations weighed inversely proportional to their innovation variance. In other words, less weight is assigned to observations from the pandemic period.^8^ Their reasoning is that the COVID-19 pandemic caused unprecedented variation in many key macroeconomic variables. The U.S. unemployment rate, for instance, increased by approximately 10 percentage points from March to April 2020, which is two orders of magnitude more than its typical monthly change. Their VAR model includes six variables for the U.S. economy: unemployment, employment and four price indices. They come to the following two conclusions. The first is that the strategy of dropping the COVID-19 observations may be acceptable for the purpose of parameter estimation. The second is that the strategy of dropping the COVID-19 observations is inappropriate for forecasting the future evolution of the economy.

An alternative possibility to deal with the COVID-19 pandemic is to relax the assumption of normal errors and allow them to follow a fat-tailed distribution as recommended by Bobeica and Hartwig (2022). They show that this strategy, assuming a multivariate t-distribution for the BVAR errors, ensures more stable parameters and forecasts for the model that includes the COVID-19 pandemic. Moreover, they demonstrate that a large standard BVAR with normal errors, but more prior shrinkage than usual, ensures stable unconditional forecasts. They emphasize the necessity to check how the COVID-19 observations affect the parameter estimates and the implied forecasts. Additionally, they point out that as new observations on the variables become available, the distortion of traditionally estimated parameters diminishes. However, they find that a simple BVAR model with normal and homoskedastic errors cannot cope with the outlier observations in COVID-19 period.

## Research methodology

In this section, we briefly outline our research methodology. First, we discuss BVAR models. Second, we describe the data used in the paper. Our monthly U.S. data cover the period from 1990:01 to 2021:11. The start date is chosen based on data availability. The end date is the most recent month available when this research was started at the beginning of 2022.

### The Bayesian VAR model

Bayesian VARs deal with the problem of overparameterization (the curse of dimensionality) of VAR models by incorporating in the estimation and forecasting process prior information. It is information beyond that contained in the data itself and takes the form of inexact prior restrictions. VAR coefficients are treated as being random variables around their prior means. The tightness of the distribution is controlled for with so-called hyper-parameters. Therefore, one must specify the form of the prior distribution and also the covariance matrix of the regression errors.^9^ Similarly, the degree of uncertainty about the long-run stochastic trends (unit roots) enters explicitly when model parameters are estimated.^10^ The Bayesian VAR literature has suggested various alternative priors over the years. Recent research has developed algorithms that do not require the VAR error covariance matrix to be fixed or diagonal (Robertson & Tallman, 1999). In our approach we use a prior distributions that belong to the conjugate normal-inverse Wishart family and we apply the shrinkage methods of Giannone et al. (2015). They advocate using prior information that shrinks large BVAR models towards parsimonious representations.

We apply the following baseline BVAR model with $n$ endogenous variables, $p$ lags, and $m$ exogenous variables.

$$\begin{aligned}{y}_{t}&={A}_{1}{y}_{t-1}+{A}_{2}{y}_{t-2}+\dots +{A}_{p}{y}_{t-p}+C{x}_{t}+{\varepsilon }_{t},\\ & \quad {\varepsilon }_{t}\sim N\left(0,\Sigma \right),\end{aligned}$$

where ${y}_{t}$ is a $n\times 1$ vector of endogenous variables, ${A}_{1}, \dots {A}_{p}$ are $p n\times n$ matrices of parameters, $C$ is a $n\times m$ matrix, ${x}_{t}$ is a $m\times 1$ vector of exogenous variables (it may contain constant terms), and ${\varepsilon }_{t}$ is a $n\times 1$ vector of errors, following a multivariate normal distribution with zero mean and covariance matrix Σ.

We define the vectors $\beta =vec\left({\left[{A}_{1},{A}_{2},\dots ,{A}_{p},C\right]}^{^{\prime}}\right)$, $y=vec\left({\left[{y}_{1},{y}_{2},\dots ,{y}_{T}\right]}^{\prime}\right)$, and $\varepsilon =vec\left({\left[{\varepsilon }_{1},{\varepsilon }_{2},\dots ,{\varepsilon }_{T}\right]}^{\prime}\right)$ for any sample size $T$. Further,

$$\begin{aligned}X& =\left(\begin{array}{ccccc}{y^{\prime}}_{0}& {y^{\prime}}_{-1}& \cdots & {y^{\prime}}_{1-p}& {x^{\prime}}_{1}\\ {y^{\prime}}_{1}& {y^{\prime}}_{0}& \dots & {y^{\prime}}_{2-p}& {x^{\prime}}_{2}\\ \vdots & \vdots & \ddots & \vdots & \vdots \\ {y^{\prime}}_{T-1}& {y^{\prime}}_{T-2}& \cdots & {y^{\prime}}_{T-p}& {x^{\prime}}_{T}\end{array}\right), \\ \overline{X }&={I}_{n}\otimes X.\end{aligned}$$

Now, the previous equation (following Dieppe et al., 2016) can be reformulated as:

$$y=\overline{X }\beta +\varepsilon ,$$

with $\beta$ a $q\times 1$ vector, where $q=n(np+m)\times 1$.$E\left({\varepsilon }_{t}{\varepsilon }_{t}^{\prime}\right)=\Sigma ,$ while $E\left({\varepsilon }_{t}{\varepsilon }_{s}^{\prime}\right)=0$ for $t\ne s$. $\Sigma$ is a $n\times n$ symmetric positive definite covariance matrix. Taking the Bayesian perspective, one needs to specify the priors for $\beta$ and for the covariance matrix $\Sigma$. The procedure is based on a modified version of Litterman’s (1986) approach. The equations are centered around a random walk with drift (cf. Equation (2) in Bańbura et al., 2010). The prior specification incorporates the belief that the more recent lags should provide more reliable information than the more distant ones. Also, own lags should explain more of the variation of the given variable than the lags of other variables in the equation. It is assumed that little is known about exogenous variables, so that the variance of these terms should be large. To account for correlation among the residuals of different variables, we use the inverse Wishart prior for $\Sigma$. Thus, in the baseline specification, we focus on prior distributions that belong to the conjugate normal-inverse Wishart family:

$$\begin{aligned}&\Sigma \sim \mathrm{IW}\left(\Psi ,\mathrm{d}\right),\\ & \beta \sim N\left(b,\Sigma \otimes\Omega \right).\end{aligned}$$

We want to compare the forecasting performance and impulse responses from small and large BVAR models. Technically a large model with about 40 endogenous variables is not possible to estimate using the proposed prior structure (Minnesota structure). Dieppe et al. (2016) calculate that in the case of a model with 40 endogenous variables, 5 exogenous variables and 15 lags, $q=24200$, which implies that each iteration of the Gibbs sampler requires the inversion of a $24200\times 24200$ matrix.^11^ This makes the process very slow and practically intractable. To partly solve this problem we use the dummy observation prior with two extensions, the sum of coefficients prior and the dummy initial observations. If we write the VAR model in its error-correction form we get:

$${y}_{t}={\left({I}_{n}-{A}_{1}-\dots -{A}_{p}\right){y}_{t-1}+B}_{1}{\Delta y}_{t-1}+\dots +{B}_{p-1}\Delta {y}_{t-p+1}+C{x}_{t}+{\varepsilon }_{t}$$

The sum of coefficient prior shrinks ${I}_{n}-{A}_{1}-\dots -{A}_{p}$ to $0$. The procedure is implemented by adding the specific dummy observations (Bańbura et al., 2010).

We use for the majority of our calculations the European Central Bank’s (ECB’s) toolbox *Bayesian Estimation, Analysis And Regression* (BEAR) version 5.1.3.^12^ We apply variable specific priors for the autoregressive coefficients. We follow Giannone et al.’s (2015) approach for the construction of the prior. If λ controls the overall tightness of the prior distribution, then for $\lambda =0$ the posterior equals the prior and the data do not influence the estimates and for $\lambda =\infty$ the posterior coincides with ordinary least squares estimates. Bańbura et al. (2010) argue that the more variables we include in the VAR model, the more $\lambda$ should shrink in order to avoid overfitting.

### Data

Monthly data for the U.S. economy are used to set up a small and a large BVAR model. The list of the variables with their descriptions, sources and transformations is in the Appendix. “LOG” below indicates that we take natural logarithms of a variable. We consider the following two VAR models:

1. The *small model* that includes: Industrial Production: Total Index (LOG_INDPRO); Consumer Price Index for All Urban Consumers: All Items in U.S. City Average (LOG_CPIAUCSL); and the Wu-Xia Shadow Federal Funds Rate.
2. The *large model* that extends the small model with additional variables:
  ° Industrial Production: Total Index (LOG_INDPRO);
  ° Total Non-Farm Payroll: All Employees, Total Nonfarm (LOG_PAYEMS);
  ° Unemployment Rate (UNRATE);
  ° Capacity Utilization Rate: Manufacturing (MCUMFN);
  ° New Privately-Owned Housing Units Started: Total Units (LOG_HOUST);
  ° University of Michigan: Consumer Sentiment Index (UMCSENT);
  ° Consumer Price Index for All Urban Consumers: All Items in U.S. City Average (LOG_CPIAUCSL);
  ° Personal Consumption Expenditures Chain-Type Price Index (LOG_PCEPI);
  ° Consumer Price Index for All Urban Consumers: All Items Less Food and Energy in U.S. City Average (LOG_CPILFESL);
  ° Personal Consumption Expenditures Excluding Food and Energy (LOG_PCEPILFE);
  ° Compensation of Employees, Received: Wage and Salary Disbursements (LOG_COMPENSATION);
  ° Crude Oil Prices: West Texas Intermediate (WTI)—Cushing, Oklahoma (LOG_OIL);
  ° Wu-Xia Shadow Federal Funds Rate;
  ° Moody's Seasoned Aaa Corporate Bond Yield (DAAA);
  ° Moody's Seasoned Baa Corporate Bond Yield (DBAA);
  ° Market Yield on U.S. Treasury Securities at 2-Year Constant Maturity (DGS2);
  ° Market Yield on U.S. Treasury Securities at 5-Year Constant Maturity (DGS5);
  ° Market Yield on U.S. Treasury Securities at 10-Year Constant Maturity (DGS10);
  ° Nominal narrow BIS effective exchange rate (NEER2);
  ° S&P 500 index (SP_CLOSE).

We choose the variables guided by previous large BVAR studies discussed in Sect. 2 and the availability of data. We note that when the Wu-Xia shadow interest rate is above 1/4 percent, it is exactly equal to the Wu-Xia model implied one-month interest rate by construction. Also, we decided to omit the real personal consumption expenditures series (PCEC96), because the series is available only from 2002:01. For the same reason we omit the real broad Bank for International Settlements (BIS) effective exchange rate and the nominal broad BIS effective exchange rate, which starts in 1994:01.

## Results

### Unit root test results

We apply standard Augmented Dickey-Fuller unit root tests only to get an indication about the dynamic persistence (memory) of shocks to variables. This helps sorting variables into slow and fast moving processes. Table 1 reveals that most of the variables are nonstationary in levels, with covariance-stationary first differences, i.e., with unit roots in levels. There are only two series which appear to be covariance stationary in levels, namely these are the unemployment rate (UNRATE) and the University of Michigan consumer sentiment (UMCSENT). We set the autoregressive *prior* coefficient ${\delta }_{i}$ equal to 1 for nonstationary variables and equal to 0.8 for stationary variables. This is based on the assumption that each nonstationary variable follows an independent random walk process, potentially with drift.

**Table 1:** Unit root tests

Series	*p* value	Lags	Maximum lags	Observations
LOG_INDPRO	0.2621	2	16	383
LOG_PAYEMS	0.5945	0	16	384
UNRATE	0.0178	0	16	384
MCUMFN	0.1099	1	16	383
LOG_PCEC96	0.8539	2	14	236
LOG_HOUST	0.6283	2	16	383
UMCSENT	0.0233	0	16	383
LOG_CPIAUCSL	0.4433	2	16	384
LOG_PCEPI	0.7102	1	16	383
LOG_CPILFESL	0.9413	12	16	384
LOG_PCEPILFE	0.7102	1	16	383
LOG_COMPENSATION	0.8235	0	16	383
LOG_OIL	0.2675	1	16	384
WU_XIA_RATE	0.2627	2	16	381
DFF	0.1579	3	16	384
DAAA	0.7950	13	16	384
DBAA	0.7319	12	16	384
DGS2	0.3353	13	16	384
DGS5	0.5544	13	16	384
DGS10	0.6759	13	16	384
REER	0.4910	2	16	332
NEER	0.3822	2	16	332
NEER2	0.2101	1	16	383
SP_CLOSE	1.0000	0	16	384

Note: We applied the standard augmented Dickey-Fuller (ADF) unit root test, with lags selected according to Akaike’s information criterion

### Impulse response functions

We follow Bańbura et al. (2010) and divide the variables into slow and fast moving. This means that the former group contains real variables and prices, while the latter consists of financial variables. The assumption that is made here is that slow-moving variables do not respond contemporaneously to a monetary policy shock and that the information set of the monetary authority contains only past values of the fast-moving variables. The ordering of the variables is from slow to fast moving variables, as listed in Sect. 3.2 under (1) for the small model and under (2) for the large model. We apply a Cholesky factorization along the lines of Dieppe and van Roye (2021) with two blocks.

**Fig. 1:**
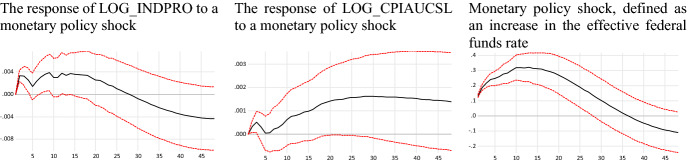
Impulse response functions for the small VAR model for the whole sample when the effective federal funds rate is used

**Fig. 2:**
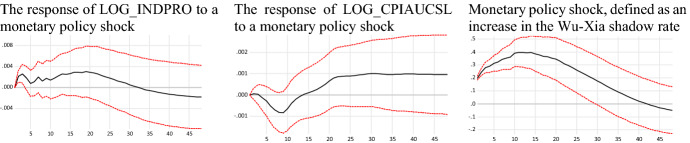
Impulse response functions for the small VAR model for the whole sample when the shadow interest rate is used

First we estimate the small VAR model with only 3 variables (industrial production, the consumer price index, and the interest rate) and 13 lags. We test whether it is better to use a federal funds effective rate (Fig. 1) or a shadow rate (Fig. 2). One may observe that the positive shadow rate impulse causes a statistically significant decrease in inflation, whereas the positive federal funds rate impulse leads to statistically insignificant reactions of prices. Also, the positive shadow rate impulse does not cause an increase in the level of industrial production as does the positive federal funds rate impulse.^13^ If we restrict the sample to end before the pandemic we end with the same conclusion that the impulse response functions are more reasonable for the shadow rate than for the federal funds effective rate. This initial experiment recommends using the shadow rate in further VAR models as the appropriate monetary policy tool.

**Fig. 3:**
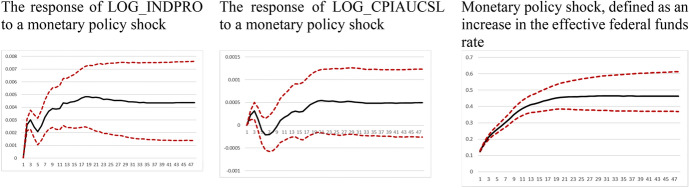
Impulse response functions for the small BVAR model with the effective federal funds rate (full sample)

**Fig. 4:**
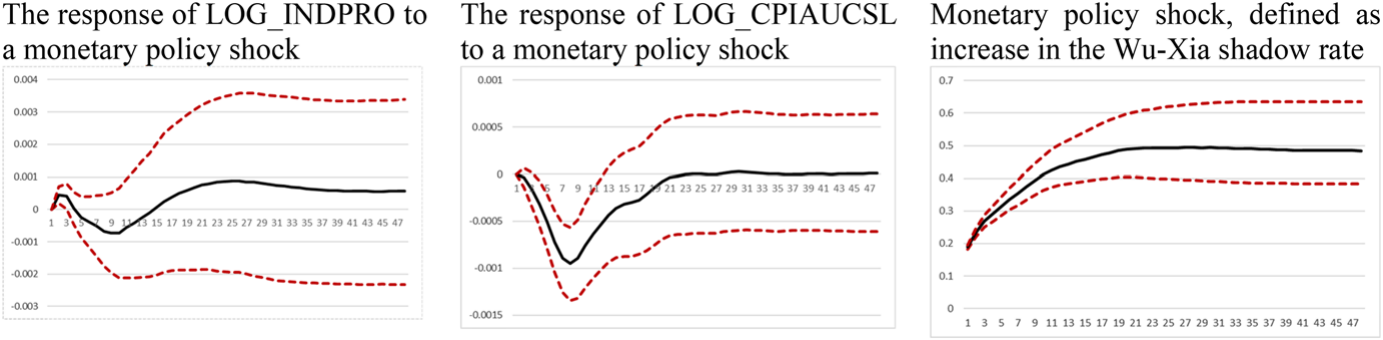
Impulse response functions for the small BVAR model with the shadow rate (full sample)

**Fig. 5:**
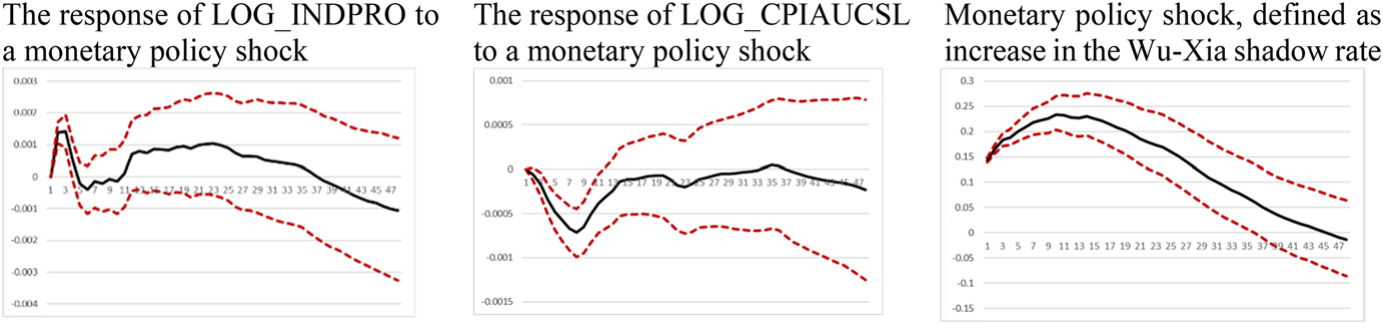
Impulse response functions for the large BVAR model

Next, we move to estimating BVAR models.^14^ In the first step we redo the experiment with the smaller model. We obtain Figs. 3 and 4. The results show that an increase in the Wu-Xia shadow rate and federal funds rate lead to a decrease in the CPI, but the response of industrial production is positive, contrary to economic theory. In the second step we estimate the large BVAR model consisting of 20 variables (Fig. 5). In this case a monetary policy impulse leads to a short lived increase in industrial production (2nd – 3rd month after the impact of the shock) and a decrease in the consumer price index (3rd – 11th month). It is worth emphasizing that the monetary policy impulse becomes statistically insignificant after 37 months for the large BVAR model, and that this is not the case for the small BVAR model. Thus, we find the impulse response functions for the large BVAR model are the most reliable.

### Forecasting

We compare first the forecasting performance of the small and large models. Lenza and Primiceri (2022) show that the BVAR model with rescaled variables produces forecasts with higher degrees of uncertainty than the BVAR model with the latest pandemic data being excluded. Next, we continue our research by doing an out-of-sample competition among the two models. We apply recursive forecast evaluation schemes, so that forecasting models are estimated on expanding windows. We calculate forecasts for 1 month, 2, 3, 6 months and 1 year horizons. We report 6 forecast evaluation criteria: the root mean squared error (RMSE), the mean absolute error (MAE), the mean absolute percentage error (MAPE), the continuous ranked probability score (CRPS), two different versions of the log score, and an adjusted Diebold-Mariano test (HLN).

#### Assessing the forecasting performance

Forecast evaluation measures can be divided into two groups, the frequentist style measures that compare the point estimates of the forecast with the actual values, and the density based measures that compare the whole posterior predictive density with the actual values.^15^ Below we present three measures from the first group and three measures from the second group.

For the chosen $y$ variable the root mean squared error of the forecast formulated in periods from ${(T}^{*}+1)$ to $(T-h$) over $h$ periods ahead is defined as:

$${RMSE}_{h}=\sqrt{\frac{1}{(T-h-{T}^{*}+1)}\sum_{t={T}^{*}+1}^{T-h}{({y}_{t+h}-{\widetilde{y}}_{t,h})}^{2}}$$

where ${\widetilde{y}}_{t,h}$ denotes the predicted value of $y$ in period $t$ over $h$ periods. Similarly, the mean absolute error of the forecast is defined as:

$${MAE}_{h}=\frac{1}{(T-h-{T}^{*}+1)}\sum_{t={T}^{*}+1}^{T-h}|{y}_{t+h}-{\widetilde{y}}_{t,h}|$$

For these two measures, a lower value indicates a better fit. The mean absolute percentage error of the forecast is defined as:

$${MAPE}_{h}=\frac{100}{(T-h-{T}^{*}+1)}\sum_{t={T}^{*}+1}^{T-h}\left|\frac{{y}_{t+h}-{\widetilde{y}}_{t,h}}{{y}_{t+h}}\right|$$

The MAPE describes the size of the error relative to the value of the variable.

Now we turn to density based measures. One may think about density forecasts as summarizing the uncertainty around point forecasts. The idea is that the predictive distribution should be such that it takes a high density at the actual data value. The continuous ranked probability score is defined as the quadratic distance between the forecast cumulative distribution function (CDF) and the empirical CDF of the observations:

$$CRPS\left(F,{y}_{t+h}\right)={\int }_{-\infty }^{\infty }{\left(F\left(x\right)-{{l}}\left(x>{y}_{t+h}\right)\right)}^{2}dx,$$

where ${{l}}(\cdot )$ denotes the indicator function, $F$ is the cumulative distribution function corresponding to the marginal predictive density for the forecast at period $t$, along with the realized value ${y}_{t+h}$ for this period. The analytical formula for $F$ is in practice not known and the Gibbs sampler is used.

The CRPS can be conceived as a penalty function sanctioning the overall distance between the distribution points and the realized values. The larger the value of the CRPS, the poorer the performance of the predictive distribution for the forecast at period $t+h$.

Next, we move to the family of log predictive scores for forecasts. The procedure of computing log predictive scores is described in the ECB’s online BEAR “technical guide”.^16^ Two base forecasting scenarios are considered. In the first case, what is evaluated is the performance of the forecasts produced by model A for variable *i* at respective periods T + 1, T + 2, … T + h. In the second case, what is evaluated is the overall performance of the forecast produced by model B for variable *i* again from period T + 1 up to period T + h. If model A has a higher average log predictive score than model B, it means that values close to the actual realizations of a time series were a priori more likely according to model A relative to model B (Giannone et al., 2015). In other words, a higher log predictive score indicates that the density forecasts produced by the proposed procedure are more accurate than those of the alternative models.

Finally, to compare the forecasting performance of the two models we build on the following Diebold-Mariano test (Diebold & Mariano, 1995). If ${\varepsilon }_{1t}$ and ${\varepsilon }_{2t}$ are the residuals for the two forecasts, and ${d}_{t}={\varepsilon }_{1t}^{2}-{\varepsilon }_{2t}^{2}$, $\overline{d }=\frac{1}{n}\sum_{i=1}^{n}{d}_{i}$ then

$${\gamma }_{k}=\frac{1}{n}\sum_{i=k+1}^{n}\left({d}_{i}-\overline{d }\right)\left({d}_{i-k}-\overline{d }\right)$$

is an estimate of the kth autocovariance and the Diebold-Mariano test statistic is equal to:

$$\frac{\overline{d} }{\sqrt{({\gamma }_{0}+2\sum_{k=1}^{h-1}{\gamma }_{k})/n}}$$

Under the assumption that the null hypothesis is true ($E\left[{d}_{i}]=0\right)$ it follows a standard normal distribution. We use the Harvey et al. (1997) correction that improves small sample properties, obtained as:

$$HLN=\sqrt{\frac{T+1-2h+h(h-1)/T}{T}}DM.$$

#### Discussion of the forecasting results

We compare the forecasting abilities of the two models (small and large) before and during the pandemic. Tables 2 and 4 compare point forecasts and Tables 3 and 5 compare density forecasts. We report the ratio between the chosen statistics for large and for small models for RMSE, MAE, MAPE, and CRPS, and the difference between the chosen statistics for small and for large model for log scores.

We present forecast evaluations for 3 variables, namely industrial production, the consumer price index, and the shadow interest rate. Tables 2 and 3 show the results for the time period before the pandemic, that is before 2020:02. Tables 4 and 5 show the results for forecasting during the pandemic, which is in the period between 2020:05 and 2021:11. In order to assess whether these models could generate reasonable forecasts during the pandemic, we decided to perform the forecasting experiment for the period after the outbreak of the pandemic. Also due to the short time that has elapsed since the outbreak of the pandemic, the assessment of forecasts applies only to a period of up to 6 months.

**Table 2:** Forecast evaluation time period before the pandemic, point forecasts

			1 month	2 months	3 months	6 months	12 months
Industrial production	Small model	RMSE:	0.004	0.006	0.007	0.010	0.015
		MAE:	0.004	0.006	0.007	0.009	0.013
		MAPE:	0.094	0.121	0.143	0.184	0.292
	Large model	RMSE:	*0.868*	*0.899*	*0.922*	*0.971*	*0.966*
		MAE:	*0.919*	*0.918*	*0.924*	*0.961*	*0.969*
		MAPE:	*0.840*	*0.851*	*0.866*	*0.920*	*0.922*
CPI	Small model	RMSE:	0.001	0.002	0.002	0.003	0.004
		MAE:	0.001	0.002	0.002	0.003	0.004
		MAPE:	0.024	0.029	0.036	0.047	0.062
	Large model	RMSE:	*0.993*	*1.050*	*1.051*	*1.026*	*1.005*
		MAE:	*0.985*	*1.029*	*1.021*	*1.005*	*1.024*
		MAPE:	*0.956*	*0.992*	*0.986*	*0.981*	*0.968*
Interest rate	Small model	RMSE:	0.114	0.181	0.243	0.423	0.817
		MAE:	0.114	0.170	0.223	0.377	0.709
		MAPE:	10,520	14,044	17,109	25,604	41,026
	Large model	RMSE:	*0.818*	*0.830*	*0.854*	*0.913*	*0.937*
		MAE:	*0.874*	*0.884*	*0.893*	*0.927*	*0.952*
		MAPE:	*0.651*	*0.677*	*0.706*	*0.768*	*0.817*

Note: Forecasting models are estimated on rolling windows, the first set of forecasts is generated using the sample 1990:01–2017:01 and the last set is generated using the sample 1992:01–2019:01. The optimal lambda parameters were found to be ${\lambda }_{1}=0.0943 \, \text{ and } \, {\lambda }_{3}=1.037$ for the small model and ${\lambda }_{1}=0.05206, {\lambda }_{3}=1.068, {\lambda }_{6}=0.02024, \, {\text{ and } \, \lambda }_{7}=0.5032$ for the large model. Values in italics indicate the ratio between the chosen statistic for the large model and the chosen statistic for the small model; thus, values above 1 mean that the small model is better than the large model

**Table 3:** Forecast evaluation time period before the pandemic, density forecasts

			1 month	2 months	3 months	6 months	12 months
Industrial production	Small model	CRPS:	0.001	0.002	0.003	0.004	0.008
		Log score 1:	3.769	3.362	3.184	2.810	2.210
		Log score 2:	3.769	7.596	11.491	23.098	46.258
	Large model	CRPS:	*1.000*	*0.979*	*0.917*	*0.979*	*0.953*
		Log score 1:	*0.071*	*0.054*	*0.076*	*0.131*	*0.070*
		Log score 2:	*− 0.052*	*0.100*	*0.310*	*0.850*	*1.803*
CPI	Small model	CRPS:	0.001	0.001	0.001	0.002	0.002
		Log score 1:	4.883	4.452	4.174	3.880	3.520
		Log score 2:	4.883	9.737	14.582	29.079	58.492
	Large model	CRPS:	*0.958*	*0.958*	*0.958*	*0.958*	*0.958*
		Log score 1:	*0.212*	*0.209*	*0.192*	*0.171*	*0.141*
		Log score 2:	*0.004*	*0.208*	*0.435*	*0.983*	*2.195*
Interest rate	Small model	CRPS:	0.048	0.072	0.094	0.154	0.265
		Log score 1:	0.412	− 0.146	− 0.482	− 1.076	− 1.915
		Log score 2:	0.412	0.896	1.431	3.114	6.320
	Large model	CRPS:	*1.105*	*1.103*	*1.086*	*1.065*	*1.067*
		Log score 1:	*− 0.065*	*− 0.139*	*− 0.145*	*− 0.116*	*− 0.187*
		Log score 2:	*− 0.061*	*− 0.052*	*− 0.035*	*0.078*	*0.228*

Note: See Table 2. The CRPS values in italics indicate the ratio between the chosen statistic for the large model and the chosen statistic for small model; thus, values above 1 mean that the small model is better than the large model. The log predictive score values in italics indicate the difference between the chosen statistic for the small model and for the large model; thus, positive values mean that the small model is better than the large model

**Table 4:** Forecast evaluation time period during the pandemic, point forecasts

			1 month	2 months	3 months	6 months
Industrial production	Small model	RMSE:	0.018	0.025	0.028	0.037
		MAE:	0.018	0.023	0.026	0.034
		MAPE:	0.408	0.508	0.575	0.743
	Large model	RMSE:	*1.300*	*1.422*	*1.554*	*1.577*
		MAE:	*1.300*	*1.416*	*1.520*	*1.471*
		MAPE:	*1.273*	*1.404*	*1.502*	*1.458*
CPI	Small model	RMSE:	0.001	0.002	0.002	0.003
		MAE:	0.001	0.002	0.002	0.003
		MAPE:	0.024	0.029	0.036	0.047
	Large model	RMSE:	*2.108*	*1.983*	*1.788*	*1.381*
		MAE:	*2.108*	*1.891*	*1.733*	*1.423*
		MAPE:	*1.992*	*1.914*	*1.718*	*1.406*
Interest rate	Small model	RMSE:	0.196	0.288	0.400	0.651
		MAE:	0.196	0.262	0.350	0.558
		MAPE:	47.401	54.650	68.105	85.844
	Large model	RMSE:	*4.023*	*4.161*	*3.919*	*2.924*
		MAE:	*4.023*	*4.291*	*4.118*	*3.109*
		MAPE:	*6.685*	*9.007*	*8.825*	*7.309*

Note: Forecasting models are estimated on rolling windows, the first set of forecasts is generated using the sample 1990:01–2020:05 and the last set is generated using the sample 1991:01–2021:05. The optimal lambda parameters were found to be ${\lambda }_{1}=0.0943 \mathrm{and} {\lambda }_{3}=1.037$ for the small model and ${\lambda }_{1}=0.01413, {\lambda }_{3}=1.072, {\lambda }_{6}=0.02056, {\mathrm{and} \lambda }_{7}=0.503$ for the large model. Values in italics indicate the ratio between the chosen statistic for the large model and the chosen statistic for the small model; thus, values above 1 mean that the small model is better than the large model

**Table 5:** Forecast evaluation time period during the pandemic, density forecasts

			1 month	2 months	3 months	6 months
Industrial production	Small model	CRPS:	0.003	0.004	0.005	0.007
		Log score 1:	0.569	0.021	0.242	0.469
		Log score 2:	0.569	2.640	5.321	13.337
	Large model	CRPS:	3.333	4.000	3.400	2.935
		Log score 1:	2.053	1.599	1.443	1.457
		Log score 2:	2.053	3.991	6.036	10.983
CPI	Small model	CRPS:	0.001	0.001	0.001	0.002
		Log score 1:	4.065	2.965	2.219	0.025
		Log score 2:	4.065	8.325	12.669	24.808
	Large model	CRPS:	*2.000*	*4.000*	*5.000*	*2.500*
		Log score 1:	*3.463*	*2.779*	*2.543*	*1.748*
		Log score 2:	*3.463*	*7.009*	*10.571*	*20.122*
Interest rate	Small model	CRPS:	0.049	0.075	0.098	0.163
		Log score 1:	−0.576	−0.948	−1.315	−1.535
		Log score 2:	−0.576	−1.148	−1.753	−3.424
	Large model	CRPS:	*4.563*	*4.775*	*4.201*	*3.049*
		Log score 1:	*−1.29*	*−1.826*	*−2.134*	*−1.881*
		Log score 2:	*−1.29*	*−2.451*	*−3.565*	*−7.397*

Note: See Table 4. For CRPS value and log score interpretations see Table 3

We test two sets of hyper-parameters, the first set is the standard values proposed in the literature (overall tightness equal to 0.1 and lag decay equal to 1), the second set is the values obtained using Giannone et al.’s (2015) procedure. For the large model and the two samples, the one that includes the pandemic and the one that ends before the pandemic, much better results were obtained using Giannone et al.’s prior. However, for the small model for the sample that ends before the pandemic no strong evidence was found that their prior is better than the standard hyper-parameter values, with results very similar for the two type of priors. Moreover, for the sample that includes the pandemic the model with standard hyper-parameter values generated better forecasts than the small model with Giannone et al.’s prior.

Forecasting models are estimated on rolling windows, for the period before the pandemic the first set of forecasts is generated using the sample 1990:01–2017:01 and the last set is generated using the sample 1992:01–2019:01. The optimal lambda parameters were found to be for the small model: ${\lambda }_{1}=0.0943 \mathrm{ and} {\lambda }_{3}=1.037;$ and for the big model: ${\lambda }_{1}=0.02144, {\lambda }_{3}=1.088, {\lambda }_{6}=0.02096, {\mathrm{and }\,\lambda }_{7}=0.5027$.

For the period during the pandemic the first set of forecasts is generated using the sample 1990:01–2020:05 and the last set is generated using the sample 1991:01–2021:05. The optimal lambda parameters were found to be: ${\lambda }_{1}=0.0943 \mathrm{and}{ \lambda }_{3}=1.037$ for the small model; and ${\lambda }_{1}=0.01413, {\lambda }_{3}=1.072, {\lambda }_{6}=0.02056, {\mathrm{and }\lambda }_{7}=0.503$ for the large model.

Based on our results so far, we are not able to conclusively confirm that the large model outperforms the small model. The forecasting performance of the two models seems to be similar. Thus, taking into account the time needed to calculate the large model, the small model might be preferable.

**Fig. 6:**
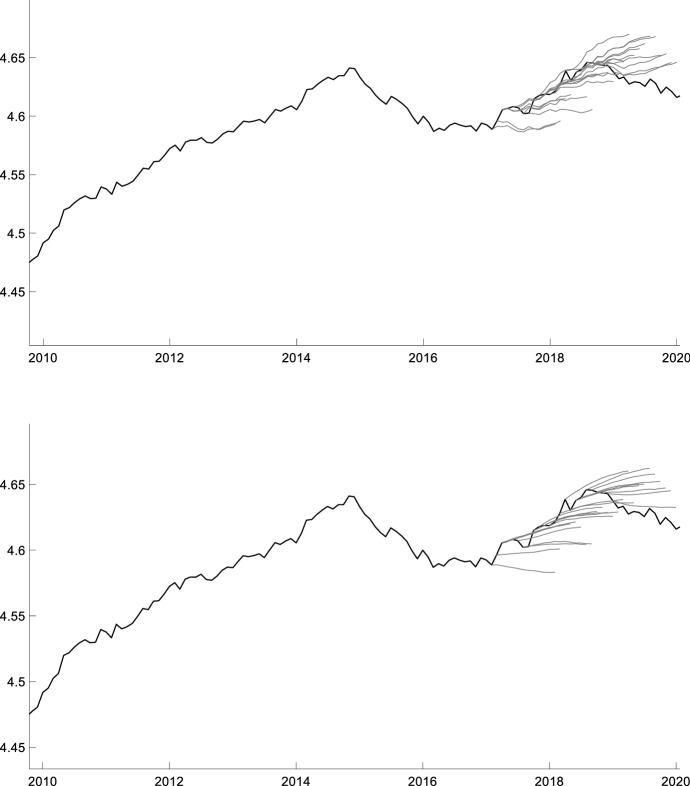
Sequential forecasts of industrial production for the small (lower panel) and the large (upper panel) model for the time period before the pandemic

Table 6 presents the results of the HLN test, a version of the Diebold and Mariano (1995) test, where the long-run variance was calculated with the Newey-West method. The results of the test show that only in 6 out of 30 cases there are statistically significant differences between the small and large models. The differences appear in the case of interest rate forecasts for 1, 2, 3, and 6 months ahead before the pandemic and in the case of the industrial production forecast for 12 months before pandemic, and lastly in the case of the interest rate forecast for 2 months during pandemic. Additionally, Fig. 6 shows sequential forecasts of industrial production before and during pandemic. One may observe that the two models indeed provide similar forecasts before the pandemic, but the picture is less clear during the pandemic.

**Table 6:** *p-values* for Harvey et al. (1997) tests to assess the statistical significance of equal predictive accuracy between small and large models

	1 month	2 months	3 months	6 months	12 months
*Before the pandemic*					
Industrial production	0.5335	0.3753	0.2645	0.2326	0.0169**
CPI	0.6903	0.8601	0.8585	0.8344	0.6231
Interest rate	0.0016***	0.0078***	0.0065***	0.0072***	0.3184
*During the pandemic*					
Industrial production	0.4755	0.5485	0.7473	0.7275	0.9748
CPI	0.5751	0.5411	0.5124	0.6913	0.8973
Interest rate	0.2737	0.0325**	0.3620	0.5504	0.9026

Note: */**/*** means rejecting the null hypothesis at, respectively, 1%/5%/10% significance levels; thus it means that the predictive accuracy of the two models is different. Also, the long-run variances were calculated with the Newey-West method

First of all, we look at the cases where the HLN test shows statistically significant differences. In the case of the interest rate forecast for 1, 2, 3, and 6 months ahead before the pandemic and in the case of the industrial production forecast for 12 months before pandemic the results presented in Table 2 indicate that the large model is better. However, the results in Table 3 are not so clear, for instance, CRPS-based statistics for the interest rate indicate that the small model is better. In the case of the interest rate forecasts for 2 months during the pandemic all measures (Tables 4 and 5) indicate that the small model is better than the large model.

Concerning the time period before the pandemic, Table 2, for example, shows that the large model outperforms the small model for the forecasts for industrial production and the shadow interest rate, according to all analyzed point forecast measures. Table 3, however, indicates that the CRPS measure is indeed lower for the large model for industrial production forecasts, but it is not for the interest rate. Log scores favor the small model for industrial production, and the large model for the interest rate. For the forecasts for the consumer price index both the traditional point forecasting measures and the density-based scores differ little and in many cases the difference is only in the third decimal place.

**Fig. 7:**
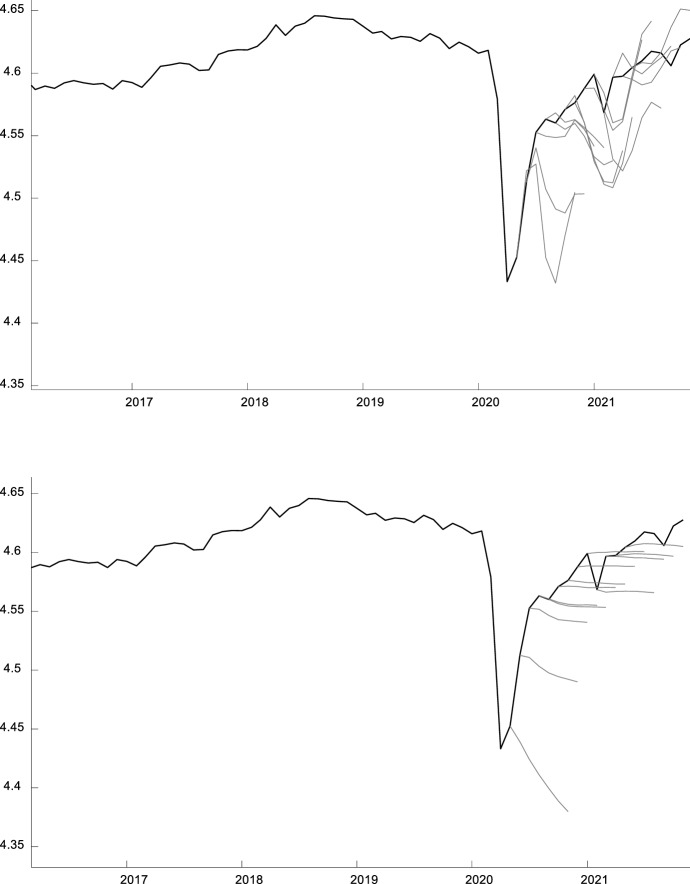
Sequential forecasts of industrial production for the small (lower panel) and the large (upper panel) model for the time period during the pandemic

In addition, if we compare the results for the pandemic period, the small model seems to outperform the large model in many cases (Fig. 7). For the forecasts for the shadow rate all error statistics are better for the small model. For industrial production and the consumer price index, only log predictive scores seem to show better performance of the large model in a few cases (see Table 5). Thus, we conclude that in this specific time period the small model is a better choice.

In general we could conclude that for the pandemic period the small model seems to perform better, and for the before-the-pandemic period the large model is slightly preferable. If we compare the forecasting performance of the models before the pandemic and during the pandemic, we observe that the forecasting performance of the models is better before the pandemic. The result holds for all three time series (i.e. industrial production, the consumer price index, and the shadow interest rate). All forecasting evaluation criteria show that forecasts produced before the pandemic (Tables 2 and 3) are more accurate than forecasts produced during the pandemic (Tables 4 and 5).

## Conclusion

We estimate the BVAR models with Giannone et al.’s (2015) prior for the United States for the recent time period from 1990:01 to 2021:11. We do not apply any data modification to the pandemic-period data. The impulse response functions that we obtain are very similar to the impulse response functions obtained for the sample that ends before the pandemic, which is in 2020:01. We test the performance of the small BVAR model (with three variables) and the large BVAR model (with 20 variables). The models seem to produce credible impulse responses. We compare their forecasting performance using traditional point forecasting measures and density-based scores in two time periods, namely before and during the pandemic.

Our results indicate that it is preferable to use the Wu-Xia shadow rate instead of the effective federal funds rate as the monetary policy tool in the VAR models. Also, our results indicate that the forecasting performance of the small BVAR model is quite similar to performance of the large VAR model. Therefore, it seems preferable to use small BVAR models instead of large BVAR models that are more difficult and time-consuming to estimate. We leave for future research some important issues: the application of conditional forecasts, optimizing for lag length, and conducting a similar study for some other economies.

## Appendix

See Table 7.

**Table 7:** Data description

	Series	Describtion	Source	Units	Transfor-mation
*Indicators of the level of economic activity*					
1	IND_PROD	Industrial Production: Total Index (INDPRO)	Federal Reserve Board	SA, 2017 = 100	Log
2	PAYEMS	All Employees, Total Nonfarm (PAYEMS)	BLS	SA, Thousands of Persons	Log
3	UNEMP	Unemployment Rate (UNRATE)	BLS	SA, % (of labor force)	None
4	CAPACITY	Capacity Utilization: Manufacturing (NAICS) (MCUMFN)	Federal Reserve Board	SA, % (of capacity)	None
5	R_PCE	Real Personal Consumption Expenditures (PCEC96)	BEA	Billions of Chained 2012 Dollars, SA	Log
6	HOUSING_STAR-TS	New Privately-Owned Housing Units Started: Total Units (HOUST)	Census Bureau	SAAR, Thousands of Units	Log
*Indicators of the level of prices*					
7	CPI	Consumer Price Index for All Urban Consumers: All Items in U.S. City Average (CPIAUCSL)	BLS	SA, 1982–84 = 100	Log
8	PCE	Personal Consumption Expenditures: Chain-type Price Index (PCEPI)	BEA	SA, 2012 = 100	Log
9	CPI_LESS	Consumer Price Index for All Urban Consumers: All Items Less Food and Energy in U.S. City Average (CPILFESL)	BLS	SA, 1982–84 = 100	Log
10	PCE_LESS	Personal Consumption Expenditures Excluding Food and Energy (Chain-Type Price Index) (PCEPILFE)	BEA	SA, 2012 = 100	Log
11	COMPENSATION	Compensation of Employees, Received: Wage and Salary Disbursements (A576RC1)	BEA	SA, Billions of Dollars	Log
12	OIL	Crude Oil Prices: West Texas Intermediate (WTI)—Cushing, Oklahoma (DCOILWTICO)	EIA / WSJ	Dollars	Log
*Interest rates*					
13	WU_XIA_RATE	Wu-Xia Shadow Federal Funds Rate	https://www.atlantafed.org/cqer/research/wu-xia-shadow-federal-funds-rate	% p.a	None
14	FF_EF_RATE	Federal Funds Effective Rate (DFF)	Federal Reserve Board	% p.a	None
15	Moody_AAA	Moody's Seasoned Aaa Corporate Bond Yield (DAAA)	Moody’s	% p.a	None
16	Moody_BAA	Moody's Seasoned Baa Corporate Bond Yield (DBAA)	Moody’s	% p.a	None
17	2Y_yield	Market Yield on U.S. Treasury Securities at 2-Year Constant Maturity (DGS2)	Federal Reserve Board	% p.a	None
18	5Y_yield	Market Yield on U.S. Treasury Securities at 5-Year Constant Maturity (DGS5)	Federal Reserve Board	% p.a	None
19	10Y_yield	Market Yield on U.S. Treasury Securities at 10-Year Constant Maturity (DGS10)	Federal Reserve Board	% p.a	None
*Exchange rate*					
20	USD_EUR	U.S. Dollars to Euro Spot Exchange Rate	Federal Reserve Board	U.S. Dollars to One Euro, Not Seasonally Adjusted	Log
22	REER	Real broad BIS effective exchange rate, Real (CPI-based), Broad Indices	BIS	Monthly averages; 2010 = 100	Log
23	NEER	Nominal broad BIS effective exchange rate	BIS	Monthly averages; 2010 = 100	Log
24	NEER2	Nominal narrow BIS effective exchange rate	BIS	Monthly averages; 2010 = 100	Log
*Additional variables*					
25	S&P 500	S&P 500 index, adjusted close price adjusted for splits and dividend and/or capital gain distributions	Yahoo! Finance	Dollars	Log
26	SENTIMENT	University of Michigan: Consumer Sentiment (UMCSENT) consumer confidence levels in the United States, before 1978 we use University of Michigan: Consumer Sentiment (DISCONTINUED) (UMCSENT1)	University of Michigan	Index 1966:Q1 = 100, Not Seasonally Adjusted	None

Note: BLS refers to the U.S. Bureau of Labor Statistics, BEA to the U.S. Bureau of Economic Analysis, WSJ to the Wall Street Journal, and BIS to the Bank for International Settlement. Also, “SA” refers to seasonally adjusted series and “Log” to taking the natural logarithm

## Footnotes

1 An advantage over so-called dynamic stochastic general equilibrium (DSGE) macroeconomic models is that VARs impose less economic theory. See Pagan and Wickens (2022) on the advantages and disadvantages of each type of modeling approach and on how VARs relate to DSGE models.

2 See, for example, Hamilton (1994).

3 See, for example, Kilian and Lütkepohl (2017) on the various approaches and their advantages and disadvantages.

4 See also Crump et al. (2021) for a recent use of this methodology, though not dealing with the pandemic.

5 In addition, they consider issues of aggregation, judgement, evaluation and validation.

6 We acknowledge that the ordering of the variables is important in the Cholesky context and different alternative orderings may produce different impulses that may or may not be economically meaningful.

7 They also use a change-point structural VAR (SVAR) model and a time-varying parameter SVAR model with sign restrictions. We prefer not to use sign restrictions for the impulse response functions and let instead the data speak for themselves.

8 Along similar lines, Carriero et al. (2022) down-weigh instead the variance of the residuals of outlier observations.

9 See, for example, Robertson and Tallman (1999) for a concise introduction to forecasting with BVARs.

10 This means that exact unit roots are not imposed on the VAR (Robertson and Tallman 1999).

11 The Gibbs sampling method draws randomly from the unconditional posterior distribution of the parameters. It is part of the class of so-called *Markov Chain Monte Carlo* Bayesian simulation methods. For an outline of the Gibbs procedure see Dieppe et al. (2016).

12 Available at https://www.ecb.europa.eu/pub/research/working-papers/html/bear-toolbox.en.html, last accessed 20 July 2022. The methodology for BEAR is developed in Dieppe et al. (2016).

13 The short-lived positive initial impulse is a case of borderline significance only.

14 It appears that the impulse response functions significantly change for the small model when the sum-of coefficient prior and the dummy observation prior are applied.

15 See, for example, Wolters (2015) and Rubaszek (2021) on ways to present competing forecast results.

16 https://github.com/european-central-bank/BEAR-toolbox, last accessed 22 July 2022.
